# Deep Brain Stimulation of the Subthalamic Nucleus Influences Facial Emotion Recognition in Patients With Parkinson’s Disease: A Review

**DOI:** 10.3389/fpsyg.2019.02638

**Published:** 2019-12-03

**Authors:** Caroline Wagenbreth, Maria Kuehne, Hans-Jochen Heinze, Tino Zaehle

**Affiliations:** Department of Neurology, Otto-von-Guericke-University Magdeburg, Magdeburg, Germany

**Keywords:** deep brain stimulation, Parkinson’s disease, emotional recognition, facial emotional expression, subthalamic nucleus

## Abstract

Parkinson’s disease (PD) is a neurodegenerative disorder characterized by motor symptoms following dopaminergic depletion in the substantia nigra. Besides motor impairments, however, several non-motor detriments can have the potential to considerably impact subjectively perceived quality of life in patients. Particularly emotion recognition of facial expressions has been shown to be affected in PD, and especially the perception of negative emotions like fear, anger, or disgust is impaired. While emotion processing generally refers to automatic implicit as well as conscious explicit processing, the focus of most previous studies in PD was on explicit recognition of emotions only, while largely ignoring implicit processing deficits. Deep brain stimulation of the subthalamic nucleus (STN-DBS) is widely accepted as a therapeutic measure in the treatment of PD and has been shown to advantageously influence motor problems. Among various concomitant non-motor effects of STN-DBS, modulation of facial emotion recognition under subthalamic stimulation has been investigated in previous studies with rather heterogeneous results. Although there seems to be a consensus regarding the processing of disgust, which significantly deteriorates under STN stimulation, findings concerning emotions like fear or happiness report heterogeneous data and seem to depend on various experimental settings and measurements. In the present review, we summarized previous investigations focusing on STN-DBS influence on recognition of facial emotional expressions in patients suffering from PD. In a first step, we provide a synopsis of disturbances and problems in facial emotion processing observed in patients with PD. Second, we present findings of STN-DBS influence on facial emotion recognition and especially highlight different impacts of stimulation on implicit and explicit emotional processing.

## Facial Emotion Processing

The ability to recognize and identify emotional cues in other people is a crucial component of human communication and interaction. In fact, deficits in emotion recognition are associated with poor social competence, interpersonal functioning, and reduced quality of life (Ruffman et al., 2008).

Especially faces are complex, concrete, and socially significant stimuli that are linked to emotional reactions (Murphy and Zajonc, 1993). Moreover, human faces hold a natural salience and attract attention more than other visual stimuli (Krebs et al., 2011). Hence, facial stimuli are widely used in studies assessing emotion processing under various conditions and in different clinical disturbances. In contrast to emotional prosodic stimuli, they are independent from language and thus offer the possibility to render comparability across different countries and languages. In fact, Ekman postulated basic emotions that are independent from literate culture and can be identified cross-culturally in facial expressions. Accordingly, pictures of emotional facial expression are a widely used tool in research on affective (neuro)sciences. The Ekman faces (Ekman and Friesen, 1976) are the oldest but still used database, but other face databases have been constituted in research studies, for instance, the Karolinska databases (Lundqvist et al., 1998), PENN Facial Discrimination Test (Erwin et al., 1992), or NimStim (Tottenham et al., 2009).

However, facial stimuli are somehow static and unnatural, as people usually encounter moving and dynamic faces in daily life. Moving facial stimuli are advantageous over static ones because they can be recognized easier and faster, especially for rather seldom and culture-defined emotional expressions as pride or defiance, presumably because photographs contain no dynamic information that helps identifying facial expressions (Kan et al., 2002). Moreover, dynamic faces lead to more widespread neural activation patterns and also arouse cortical regions associated with higher social relevance (Sato et al., 2004; Trautmann et al., 2009).

To a certain degree, the processing of facial stimuli underlies some attentional biases. Fearful, angry, or generally threatening faces are detected faster than neutral ones (Ishai et al., 2004; Susa et al., 2012) in accordance with the evolutionary point of view of a faster processing of threatening or possibly life-endangering stimuli. A facilitation of visual search tasks to identify fear-related pictures among fear-irrelevant ones was demonstrated (Öhman and Mineka, 2001), as well as slower attention disengagement from angry faces compared to neutral or happy ones (Fox et al., 2002). The “automatic vigilance” hypothesis implies that people tend to focus their attention preferentially on negative stimuli and can also rather difficult dissolve it from them (Wentura et al., 2000; Öhman and Mineka, 2001). However, there are also hints that postulate an advantageous processing of positive stimuli, that is, words and facial expressions (Kuchinke et al., 2005; Hofmann et al., 2009; Kissler and Koessler, 2011; Wagenbreth et al., 2014). Thus, decoding and interpreting emotions displayed in facial expressions, especially those with negative valence, play a fundamental role in human interactions.

Another interesting factor when investigating facial emotion processing is the task type used in a study. Emotion processing can be assessed by identification tasks in which participants are requested to select an appropriate label for a given emotion. In contrast, in discrimination or matching tasks, one has to judge whether or not two faces express the same emotion. These different forms of emotion recognition also reflect different and successive processing stages. Both recognition processes occur relatively early after stimulus presentation. But while the perception if two faces are identical happens rather automatic and unconsciously, that is, in an implicit way, identification tasks require additional knowledge and conscious deliberation of facial expression information as they rather involve explicit emotional processing. Hence, implicit emotional processing refers to the automatic and involuntary processing of emotional stimuli that a person is confronted with (e.g., pictures or voices). In contrast, explicit processing is the downstream process afterward that requires cognitive abilities (e.g., concentration, attention) to process and classify these given emotional stimuli.

Both forms of emotion processing may be differentially compromised in patients with Parkinson’s disease (PD), as will be shown in *Recognition of Facial Emotion in Parkinson’s Patients* of this review. Subthalamic stimulation may further have the potential not only to influence emotional recognition out of facial expressions but also to affect implicit and explicit emotional processing in a distinct way. In the following sections of this review, we will point out findings on facial emotional processing in PD patients. Then, we will focus on how subthalamic stimulation may impact those processes and, finally, discuss methodological differences between studies.

## Recognition of Facial Emotion in Parkinson’s Patients

Parkinson’s disease is a neurodegenerative disease characterized by increasing motor impairments, such as tremor, rigidity, and bradykinesia due to a depletion of dopaminergic neurons in the striatum. While PD patients occasionally suffer from motor impairments in daily life, a spectrum of concomitant non-motor symptoms including changes in mood, impulsivity, and other neuropsychiatric aspects, as well as deficits in cognitive and emotional processing have the potential to considerably influence subjective well-being and quality of life in patients (Limousin et al., 1998; Romito et al., 2002; Ballanger et al., 2009; Abbes et al., 2018).

In the 1980s, it was first suggested that PD patients also suffer from a selective impairment in facial emotion recognition (Beatty et al., 1989; Blonder et al., 1989). Since then, this observation has been replicated in a vast majority of subsequent studies, showing deficient facial emotion recognition in PD patients when compared to healthy controls (HC) (e.g., Jacobs et al., 1995; Kan et al., 2002; Sprengelmeyer et al., 2003; Yip et al., 2003; Suzuki et al., 2006; Lawrence et al., 2007; Clark et al., 2008). Accordingly, reviews and extended meta-analyses demonstrated deficits in facial emotion processing and outlined potentially biasing and correlated factors between studies like visuospatial or cognitive deficits, disease severity, or mood disorders of patients among others (Assogna et al., 2008; Gray and Tickle-Degnen, 2010; Argaud et al., 2018). In particular, emotion recognition of faces was reported to be affected in all basic emotions, but most deficits concerned the recognition of negative emotions (Argaud et al., 2018). In contrast, however, other studies did not reveal any deterioration in emotion processing in PD (Madeley et al., 1995; Adolphs et al., 1998; Breitenstein et al., 1998; Pell and Leonard, 2005).

It was suggested that emotion recognition deficits in PD were associated with influences of dopaminergic medication, but results are inconsistent. Gray and Tickle-Degnen (2010) reported emotion perception to be largely unaffected by medication status; other studies supported this assumption (Yip et al., 2003; Péron et al., 2009; Cohen et al., 2010; Roca et al., 2010; Enrici et al., 2015). Contrastingly, several publications demonstrated beneficial effects of L-Dopa on emotion recognition (Tessitore et al., 2002; Sprengelmeyer et al., 2003; Delaveau et al., 2010), suggesting that L-Dopa partially restores amygdala response but in dependence of disease progression (Delaveau et al., 2009). In fact, in the early disease stages, mesocorticolimbic pathways are described to be relatively spared compared with the motor pathway (Braak et al., 2004), and L-Dopa needed to improve motor symptoms would at the same time overdose mesolimbic projections to subcortical structures involved in emotion processing like the amygdala, leading to detrimental effects in emotion perception, whereas in later disease stages, these effects would be beneficial.

Interestingly, studies consistently demonstrating deficient emotion recognition in PD patients generally assessed explicit emotional processing by explicit evaluation and naming of the emotional value of facial expressions (Yip et al., 2003; Kan et al., 2004; Schröder et al., 2006; Ibarretxe-Bilbao et al., 2009; Péron et al., 2015; Xi et al., 2015). Here, diminished recognition was demonstrated for specific emotions such as anger (Sprengelmeyer et al., 2003; Dujardin et al., 2004; Lawrence et al., 2007; Clark et al., 2008), surprise (Clark et al., 2008), fear (Kan et al., 2002; Sprengelmeyer et al., 2003), and disgust (Kan et al., 2002; Dujardin et al., 2004; Suzuki et al., 2006; Assogna et al., 2008).

In contrast, implicit emotional recognition abilities in PD have received rather limited attention so far, and thus, implicit processing deficits are less proven. Wieser et al. (2006) tested implicit and explicit emotion processing by means of event-related potentials in response to affective pictures. The authors reported preserved early implicit processing but blunted explicit emotional responses in PD patients, supporting the view that the elementary–implicit–reaction to an emotion is independent from the conscious response to it. Hence, the emotional salience detection from faces may be compromised in PD while early perceptual and structural face processing appears to be intact. Two further studies investigated implicit emotional processing of pictorial and verbal affective stimuli but did not test for emotional facial expressions (Castner et al., 2007; Borg et al., 2012). In accordance, they also reported preserved implicit emotional processing in PD patients. In a previous study, we assessed explicit as well as implicit emotional processing in PD (Wagenbreth et al., 2016). For this purpose, we investigated emotion recognition of circumscribed facial information, for example, emotional states portrayed only in the eye region of a face, in an affective priming paradigm. We found largely preserved implicit (i.e., automatic and unconscious) emotional processing of facial expressions in PD patients, as shown by a preserved sensitivity to emotional priming; however, with a specific altered processing of disgust- and happiness-connoted stimuli. In contrast and consistent with the literature, explicit emotional processing was considerably impaired for facial stimulus material in PD.

Hence, PD compromises explicit emotional processing in general, as was shown for semantic, pictorial, and facial stimuli (Castner et al., 2007; Borg et al., 2012; Wagenbreth et al., 2016), with a particular emphasis on facial emotion recognition due to its significance in social contexts. However, the ability to be implicitly sensitive to emotional content seems to be largely spared by the disease, especially for facial stimuli.

Yet, the presented studies on facial emotional processing in PD differ with respect to several methodological, clinical, and patients’ individual factors and thus lack direct comparability. Because of this high variability in studies, several confounding factors need to be considered. In their meta-analysis, Gray and Tickle-Degnen (2010) pointed out seven potential moderators of emotional facial processing in PD that might be associated with inconsistent results of previous studies. Three of these postulated moderators concerned methodological aspects referring to the task (stimulus modality, task type, emotion displayed), and the other four relate to the patients themselves (medication status, motor disability, depression status, executive functions, and visuospatial abilities). The authors also stressed the role of potential working memory constraints during the task. Argaud et al. (2018) further emphasized the relevance of possible interactions between facial emotion impairments and mood disorders.

Functional neuroimaging studies investigating the neural basis of emotion recognition in healthy human subjects have proposed both the ventral striatum and the amygdala to be involved in processing of negative emotions like fear and disgust (Morris et al., 1996; Phillips et al., 1998). These structures consistently receive afferents from the dopaminergic neurons of the mesolimbic ventral tegmental area, which is known to degenerate in PD. The amygdala is supposed to represent an essential factor of the emotional face processing impairments in PD (Braak et al., 1994; Harding et al., 2002; Yoshimura et al., 2005). In a functional imaging study, Kipps et al. (2007) reported that the amygdala volume correlated with the ability to recognize happy facial expressions. In fact, projections from the amygdala reach the striatum, and the amygdala itself undergoes severe pathological changes during the course of PD (Braak et al., 1994). However, PD-related amygdala involvement seems to be unrelated to cognitive impairments (Braak et al., 1994), indicating that emotion processing associated with the amygdala might be spared even with ongoing PD pathology. Other attempts to explain deviant emotion processing in PD thus focus on the involvement of dopamine in emotional processing. Ongoing dopamine depletion of the mesolimbic pathway leads to a dysfunction of the limbic loop, which in turn links the basal ganglia to the orbitofrontal cortex (OFC), which leads to an impaired neuronal processing in the limbic projection area (Alexander et al., 1986; Cummings, 1993). Furthermore, the involvement of the striatum and specifically the insula in the processing of disgust was postulated (Sprengelmeyer et al., 1996; Phillips et al., 1997; Calder et al., 2000). The insula is highly interconnected with the basal ganglia and cortical regions, interacts with multiple brain networks (Chikama et al., 1997; Fudge et al., 2005), and is one of the first cortical regions that is pathologically affected in PD (Braak et al., 2006). Lesions of the insula impair the recognition of facial emotions (Calder et al., 2000), and the loss of normal metabolic activity in insular neurons in PD has been associated with blunted emotions in PD patients (Wieser et al., 2006; Robert et al., 2012). Finally, subcortical activation of the thalamus, putamen, and basal ganglia during emotional processing was observed in several functional MRI (fMRI) studies (Morris et al., 1999; Sander et al., 2005; Cheung et al., 2006), demonstrating involvement of the basal ganglia in emotion processing.

Another approach to explain diminished emotion processing in PD patients has been discussed. According to the *simulation theory of emotions*, some authors assume the contribution of motor impairments to the emotion recognition deficit (Goldman and Sripada, 2005; Niedenthal, 2007). PD-inherent symptoms like facial amimia and dysprosody have been discussed as being partially responsible for the patients’ inaccuracy in emotion recognition, but other investigations report contradictory results (see Assogna et al., 2008, for a review).

## Influence of Subthalamic Stimulation on Facial Emotion Processing in PD

### Method

We conducted a detailed search of literature with the aim of reviewing all of the relevant papers on STN-DBS influence on facial emotion processing in PD patients. We searched PubMed services (April 2019) with the following keywords: *Parkinson’s disease, deep brain stimulation, subthalamic stimulation, emotion recognition*, *emotion processing*, *face*, and *facial expression*. We also hand-searched relevant journals and examined the references of retrieved key articles to find possible further publications regarding facial emotion processing. This review particularly focused on reported comparisons between both DBS conditions (ON and OFF) and thus aims to report within-group differences between both measure conditions. Furthermore, only those publications were chosen that explicitly reported patients’ performances on emotion recognition out of faces or facial stimuli, at least as a subtask among other tests. Articles were restricted to the English language and were published between 2003 and 2019. Nineteen publications fulfilled the abovementioned criterions and were identified as being relevant to the question of how STN-DBS influences facial emotion recognition in patients (Table 1).

**TABLE 1:** Influence of STN-DBS on recognition of emotional facial expressions in PD patients.

	***N***	**Disease duration**	**L-dopa therapy**	**Experimental design**	**Task**	**Emotional stimuli**	**Results**
Schneider et al., 2003	12 PD	17.0 ± 6.3 years	Yes	Med only; Med-off/DBS-OFF; Med-off/DBS-ON	Discrimination	PENN facial discrimination test (Erwin et al., 1992)	• No stimulation effects on emotion discrimination; but mood-enhancing effect and improved emotional memory under DBS
Schroeder et al., 2004	10 PD	16 ± 3.1 years	Yes (except 1)	DBS-OFF; DBS-ON	Identification	FEEST (facial expressions of emotions: stimuli and test) (Young et al., 2002)	• Reduced recognition of angry expressions during DBS • No changes in facial emotion processing for all other emotions
Dujardin et al., 2004	12 PD, 12 HC	13 ± 2.5 years	Yes	DBS-OFF (pre-surgically); DBS-ON (3 months after surgery)	Intensity rating of expressions on emotion rating scales	Emotional facial expressions from Hess and Blairy (1995)	• General impairment in facial emotion decoding after surgery in 9/12 patients • Significant post-operative impairment for sadness, anger, and a trend for disgust
Biseul et al., 2005	15 PD (post-operative group), 15 PD (preoperative group), 15 HC	15 ± 6.2 years	Yes	DBS-OFF; DBS-ON	Identification	Ekman and Friesen, 1976	• Specific impairment to recognize fear in the tested post-operative group compared to preoperative group and HC; but no difference between DBS-ON and DBS-OFF within this group for all facial expressions
Geday et al., 2006	10 PD, 22 HC	13 ± 3.2 years	Yes	DBS-OFF; DBS-ON	PET study: no task, patients were instructed to look at pictures; After 1 week evaluation of “pleasantness”	EPS (Empathy Picture System)	• Inhibited emotional activation of the right fusiform gyrus under DBS • DBS raised emotional activation of the anterior cingulated and lowered activity of the putamen
Berney et al., 2007	15 PD	12.0 ± 6.0 years	Yes	Med-off/DBS-ON; Med-off/DBS-OFF; Med-on/DBS-OFF; Med-on/DBS-ON	Matching	Computerized task consisting of pairs conveying the same or different emotion	• Changes in mood core dimensions under stimulation, but stable emotion discrimination processing
Drapier et al., 2008	17 PD	11.8 ± 2.6 years	Yes	DBS-OFF (3 months pre surgery); DBS-ON (3 months post-surgery)	Identification	Ekman and Friesen, 1976	• Impaired fear and sadness recognition after STN-DBS • Worsened apathy scores after DBS
Le Jeune et al., 2008	13 PD, 30 HC	10.9 ± 2.2 years	Yes	DBS-OFF (3 months pre surgery); DBS-ON (3 months post-surgery)	Identification	Ekman and Friesen, 1976	• Selective reduction of fear recognition under stimulation • Correlation between reduced glucose metabolism in the right orbitofrontal cortex and reduced fear recognition
Péron et al., 2010a	24 PD, 20 untreated PD (pathological control group), 30 HC	11.9 ± 2.5 years	Yes	DBS-OFF (3 months pre surgery); DBS-ON (3 months post-surgery)	Identification	Ekman and Friesen, 1976	• Impaired fear and sadness recognition after STN-DBS
Péron et al., 2010b	13 PD, 13 HC	10.5 ± 3.6 years	Yes	DBS-OFF (3 months pre surgery); DBS-ON (3 months post-surgery)	Identification	RMET (Baron-Cohen et al., 1997)	• Reduced Emotion score for patients under DBS but unchanged results in gender attribution task
Mondillon et al., 2012	14 PD, 14 HC	12.3 ± 0.7 years	Yes	Med-off/DBS-ON; Med-off/DBS-OFF; Med-on/DBS-OFF; Med-on/DBS-ON	Identification/Categorization	Karolinska emotional faces database (Lundqvist et al., 1998)	• Decreased recognition of disgust under DBS (and Med-off) • No impairment in emotion recognition observed when both therapies (Med and DBS) were “ON” • Combined administration of Med and DBS has more benefit on facial emotion recognition than the separate administration of therapies alone
Aiello et al., 2014	12 PD, 13 HC	10.9 ± 4.1 years	Yes	Med-off/DBS-ON; Med-off/DBS-OFF; Med-on/DBS-OFF; Med-on/DBS-ON	Discrimination, Intensity rating of expressions on emotion rating scales	NimStim set (Tottenham et al., 2009)	• Process of DBS surgery (microlesions) reduced patients’ performance on discrimination task independently of stimulus type • No changes in facial emotion recognition, stable performance on facial emotion discrimination after 4 months post-surgery, except for disgust
Albuquerque et al., 2014	30 PD	15.9 ± 7.0 years	Yes	DBS-OFF (before surgery); DBS-ON (1 years after surgery)	Matching, Identification	CATS (Comprehensive Affect Testing System) (Froming et al., 2006)	• No changes in discrimination and naming of emotional faces under DBS
Mermillod et al., 2014	14 PD, 14 HC	12.4 ± 0.7 years	Yes	Med-off/DBS-ON; Med-off/DBS-OFF; Med-on/DBS-OFF; Med-on/DBS-ON	Identification	Ekman and Friesen, 1976	• Lower overall recognition rate for high spatial frequency emotional faces under DBS • No effect of DBS on recognition of either broad or low spatial frequency faces
McIntosh et al., 2015	9 PD, 7 untreated PD (receiving Med), 23 elderly HC, 21 young HC	NA	Yes	Med only; DBS-OFF; DBS-ON	Identification	RMET, TASIT (Awareness of Social Inference Test, face + voice), (McDonald et al., 2003)	• Neither therapy type (Med or DBS + Med) nor therapy state (ON/OFF) changed emotion recognition
Irmen et al., 2017	11 PD, 11 HC	11.5 ± 4.2 years	Yes (except 1)	DBS-OFF; DBS-ON	Emotional Stroop Task	2D Facial Emotional Stimuli dataset (Erwin et al., 1992)	• No conflict-induced slowing under DBS • Valence bias affecting conflict-induced reaction time slowing under DBS-OFF
Enrici et al., 2017^∗^	18 PD, 20 untreated PD (receiving Med), 20 HC	12.6 ± 3.0 years	Yes	Med only; Med-on/DBS-ON	Identification	Ekman and Friesen, 1976; RMET	• Comparisons only for DBS versus HC, no comparison between DBS-ON and DBS-OFF • No impairment in facial emotion recognition and in affective Theory of Mind in DBS patients compared to HC
Martínez-Fernández et al., 2018	16 PD, 16 HC	10.6 ± 3.4 years	Yes	Med-off/DBS-ON; Med-off/DBS-OFF; Med-on/DBS-OFF; Med-on/DBS-ON	Emotional Stroop Task	Karolinska emotional faces database (Lundqvist et al., 1998)	• For fearful faces, emotional Stroop effect was higher under dopaminergic treatment than under DBS • Both treatments did not modulate Stroop effect • EEG: L-Dopa but not DBS increases the amplitude of the event-related potential N170
Wagenbreth et al., 2019	14 PD	11.7 ± 4.4 years	Yes (except 2)	DBS-OFF; DBS-ON	Affective priming paradigm, identification	Ekman and Friesen, 1976	• DBS affected explicit emotional processing more than implicit processing • DBS selectively diminished explicit processing of disgust but had an ameliorating effect on discriminating fear stimuli

*^∗^No within-group comparison; PD: Parkinson’s disease patients; HC: healthy controls; Med: medication; DBS: deep brain stimulation.*

### Facial Emotion Recognition Under STN-DBS

#### Anatomical and Structural Correlates of Emotional Processing Under STN-DBS

Subthalamic stimulation has been variously proven to be a helpful therapeutic tool to improve motor disturbances in PD (Frank et al., 2004; Hershey et al., 2004, 2010) but has also been shown to influence cognitive domains, such as executive functions and language (Fasano et al., 2010; Wagenbreth et al., 2015; Zaehle et al., 2017). It further plays a central role in the regulation of limbic and emotional/affective functions (Temel et al., 2005; Mallet et al., 2007). The STN is part of the basal ganglia, which are interconnected to specific motor, cognitive, and limbic cortical regions through partially closed and sequentially arranged circuits (Alexander and Crutcher, 1990; Parent and Hazrati, 1995). The STN is thus closely connected with the ventral striatum and ventral pallidum, as well as to limbic cortical areas such as the anterior cingulated cortex and the OFC; regions that are known to play a major role in the recognition of emotions from faces and voices (Adolphs, 2002; Wildgruber et al., 2006) and from emotional prosody (Sander et al., 2005). DBS of the STN modifies dopaminergic transmission in the basal ganglia and thus affects the limbic, associative, and motor network circuits (Temel et al., 2005; Mallet et al., 2007). Considering the anatomic overlap of the functional areas of the STN (Haynes and Haber, 2013), stimulation of the dorsolateral motor region of the STN may as well have effects on the mesolimbic basal ganglia loops (Castrioto et al., 2014), leading to effects in emotion processing, for instance. In this context, STN-DBS has been described to occasionally cause neuropsychiatric effects like mood disturbances or depression (Berney et al., 2002; Krack et al., 2003). A relation between depression and emotion processing deficits has been suggested, but analyses yielded different results concerning possible interactions, suggesting emotion processing deficits not to be a consequence of depression or mood disturbances (Gray and Tickle-Degnen, 2010). Schneider et al. (2003) investigated the relation between mood induction and emotion recognition under STN-DBS and found a mood-enhancing effect as well as intensified emotional experience under stimulation but no changes in emotion recognition out of faces. The authors concluded that emotional recognition and categorization may involve other brain areas than those involved in the sole emotion memory or emotion experience (Schneider et al., 2003).

Generally, studies investigating the effects of STN-DBS on facial emotion perception in PD demonstrated rather heterogeneous results, and either covered unchanged emotion recognition of facial expressions under DBS (Schneider et al., 2003; Berney et al., 2007; Albuquerque et al., 2014; McIntosh et al., 2015) or worsening of explicit discriminating emotional faces under stimulation (Geday et al., 2006; Péron et al., 2010a, b). Precisely, a tendency for DBS to cause deficits in facial discrimination of especially negative emotions like disgust (Mondillon et al., 2012; Aiello et al., 2014; Wagenbreth et al., 2019), anger (Schroeder et al., 2004), sadness (Drapier et al., 2008), and fear (Drapier et al., 2008; Mondillon et al., 2012) was observed.

Aiello et al. (2014) found diminished disgust discrimination abilities under DBS for facial expressions and emotional prosodic stimuli in PD patients but emphasized that impaired disgust recognition was prominent also before DBS implant in patients, which is in line with other studies (see *Recognition of Facial Emotion in Parkinson’s Patients*). Impaired processing of disgust is thus related to the neurodegenerative disease itself rather than just an impact of STN-DBS (Mondillon et al., 2012; Aiello et al., 2014). Still, there seems to be consensus that disgust processing in faces and also in other stimulus modalities (e.g., prosody) even worsened under STN-DBS (Vicente et al., 2009; Mondillon et al., 2012; Aiello et al., 2014). Results concerning other negative emotions are not that conclusive. For instance, while some studies could demonstrate a clear deterioration of fear recognition under stimulation (Drapier et al., 2008; Le Jeune et al., 2008; Péron et al., 2010a), others failed to show any worsening of fear processing under DBS (e.g., Schroeder et al., 2004; Aiello et al., 2014; McIntosh et al., 2015).

There are different attempts to explain findings of reduced facial emotion processing of especially negative emotions. Previous investigations found activity modulations in brain regions associated with emotional processing during STN stimulation. PET studies in patients showed activity changes in non-motor areas of the associative and limbic circuits during STN-DBS and thus contribute to the findings of the central role the STN holds in motor, cognitive, and limbic basal ganglia circuits (Schroeder et al., 2002, 2003; Hilker et al., 2004). Using local field potentials, Kühn et al. (2005) found limbic activation of the STN in response to emotionally arousing pictures and proposed that this might be a reason for altered affect in PD patients. Several authors hence proposed impaired facial emotion recognition to be a result of a limbic dysfunction induced by STN-DBS (Dujardin et al., 2004; Biseul et al., 2005; Drapier et al., 2008). Le Jeune et al. (2008) demonstrated a correlation between impaired fear recognition under STN-DBS and a decrease in glucose metabolism in the right OFC. In their positron emission tomography (PET) study, Geday et al. (2006) proposed that the stimulation of the STN would inhibit regional blood flow rate activity in the right lateral fusiform gyrus, an area that is generally activated by emotional facial expressions, leading to altered emotion perception in faces.

#### Implicit and Explicit Emotional Processing Under STN-DBS

Most studies investigating DBS influence on emotion recognition clearly concentrated on one form of emotion processing only. Whereas nearly all studies in this review measured the explicit identification of emotional facial expressions, Geday et al. (2006) rather investigated the emotional reaction of patients to the pictures displayed. Patient’s general recognition of facial expressions was significantly altered by STN stimulation, whereas emotional assessment *per se* appeared to be unaffected during DBS because patients showed no significant perception changes for DBS-ON or OFF. The authors assumed that patients first needed to identify the emotional valence of a picture to subsequently assess an empathetic reaction to this picture. Geday et al. (2006) hence referred to different aspects of emotional processing, pointing out intact implicit, but impaired explicit emotional processing of facial expressions under DBS. This is in line with a study measuring implicit and explicit emotional lexical-semantic processing, suggesting that basal ganglia-thalamocortical circuits are likely not to be involved in the automatic (implicit) activation of emotion evaluations (Castner et al., 2007). Basal ganglia-thalamocortical activation would be necessary if cognitive-driven decisions are requested.

This notion was seized further in the study by Wagenbreth et al. (2019), in which, for the first time, DBS impact on implicit as well as explicit emotional processing in PD patients was examined. In an affective priming paradigm, the authors used emotional words and emotional circumscribed facial regions, that is, human eyes displaying different emotions. DBS affected explicit emotional processing more than implicit processing and had a considerable diminishing effect on the processing of disgust stimuli, but even improved explicit processing of fear stimuli. This study supported the assumption of the varying involvement of the basal ganglia, depending on demanded conscious or automatic stimuli perception and processing, and further stressed the existence of different neural mechanisms for different emotional expressions. As already described in *Recognition of Facial Emotion in Parkinson’s Patients*, referring to studies assessing emotion processing in non-stimulated PD patients, the classification of analyses in implicit and explicit emotional processing is rather neglected in the following presented studies. Most studies in this review report data of explicit processing only. Hence, a purpose and challenge for future studies lie in the investigation of both aspects combined.

#### Influence of Medication Status

One possible confounder that may explain differences between the reported studies is additional medication intake despite STN-DBS in patients. In all presented studies, patients received additional dopaminergic medication, which was applied supplementary to DBS (except for single patients). Some publications, however, tried to filter out L-Dopa-associated effects on facial recognition performance under DBS and deployed an experimental setting with alternating testing conditions for medication and DBS: (1) Med OFF/DBS OFF; (2) Med OFF/DBS ON; (3) Med ON/DBS OFF; (4) Med ON/DBS ON (Berney et al., 2007; Mondillon et al., 2012; Aiello et al., 2014; Mermillod et al., 2014; Martínez-Fernández et al., 2018). Two other studies reported data for facial recognition in three different conditions: for DBS ON and OFF and for medicated patients only (Schneider et al., 2003; McIntosh et al., 2015). Whereas Schneider et al. (2003) tested the identical patient group with medication only and with DBS only to ensure direct comparability, the medication group in the study of McIntosh et al. (2015) consisted of different patients from those in the tested DBS patient group.

In general, emotion recognition was described to be unaffected by medication status, and this was even found for patients not yet treated with STN-DBS (Roca et al., 2010; McIntosh et al., 2015). However, different studies demonstrate that the interaction between dopaminergic medication and STN-DBS offers the best results in facial emotion recognition (Mondillon et al., 2012; Martínez-Fernández et al., 2018). To explain this finding, modifications to the non-motor basal ganglia-thalamocortical circuitry and to the emotional functions of the OFC and amygdala through DBS and L-Dopa medication have been proposed. The interaction between L-Dopa and STN-DBS plays a crucial role for patients, since in most cases, dopaminergic medication intake is continued despite DBS implant in patients. In fact, all studies presented in Table 1 report additional levodopa intake besides STN-DBS. L-Dopa could overdose the mesolimbic projections toward the amygdala and OFC and thus lead to altered amygdala activation in response to emotion perception (Delaveau et al., 2009; Vicente et al., 2009; Aiello et al., 2014). DBS would compensate this overactivation by decreasing OFC activity and thereby restoring the necessary OFC-amygdala interaction (Mondillon et al., 2012). In turn, L-Dopa would compensate for the decreasing effect DBS has on the OFC and amygdala, which may explain facial recognition improvement when both therapeutic measures are “ON.” Another explanation was given by Vizcarra et al. (2019), who suggested therapeutic synergism of the effects of L-Dopa and DBS. In their meta-analysis, they found that the combined effect was greater than either treatment alone, while both alone lessened motor severity in patients to a similar effect.

Moreover, neurophysiological studies could show differing regional brain activation of areas associated with facial recognition, depending on therapy means. In their EEG study, Martínez-Fernández et al. (2018) demonstrated that the peak of the event-related potential N170, which is thought to represent facial integration and to be modulated by facial emotional content, was increased by levodopa, but not by DBS. While levodopa induced stronger activity in the right fusiform gyrus that generated the N170, STN-DBS hypoactivated this region. Geday et al. (2006) used PET to compare functional activations of brain regions when processing emotional facial expressions. They found inhibited regional blood flow rates in the right fusiform gyrus under DBS, which was not prominent when stimulation was switched OFF. STN-DBS did not change fusiform reaction to emotional expressions but raised the emotional activation of the anterior cingulate and lowered the activity of the putamen.

#### Methodological and Experimental Differences Between Studies

In general, it is difficult to draw reliable conclusions for distinct results of the single publications given the methodological and experimental differences between studies. However, one recurring finding throughout studies is that the impairment in emotion recognition in patients could not be attributed to secondary variables, such as depression, anxiety, cognitive declines, or visuospatial deficits (Dujardin et al., 2004; Schroeder et al., 2004; Drapier et al., 2008; Albuquerque et al., 2014). Hence, emotion recognition deficits are to be already associated with disease-specific mechanisms. For instance, the insula and amygdala have been shown to be involved in the processing of facial emotions (Sprengelmeyer et al., 1996; Calder et al., 2000) but to be involved in pathological changes due to PD, as described in *Recognition of Facial Emotion in Parkinson’s Patients* of this review.

Moreover, the impairment in facial emotion recognition seems to selectively concern negative emotions, while findings on positive emotions are rather scarce or non-existing. Yet, one has to keep in mind that studies examined far more negative (fear, anger, sadness, disgust) than positively valenced emotions. Actually, only happiness serves as a positive emotion throughout all studies; some publications also included surprise, whose valence is difficult to define as positive or negative without explanatory context due to its ambiguity. Also, happiness can have distinct reasons and thus different implications and consequences. Hence, there exists a bias concerning the frequency and fluency of negative versus positive emotions because negative emotions are far more differentiated than positive ones. From the evolutionary point of view, negative emotions imply greater functional value than positive ones because they help ensure “surviving” and coping in negative situations–and are thus more noticeable than positive emotions in daily life. In accordance, the “*angry face advantage*” postulates that angry faces are detected faster than other emotional and neutral faces (LoBue, 2009). But contrastingly, happiness seems to be easily recognized from facial expressions, which usually leads to ceiling effects in some studies (Kan et al., 2002). The described effect of a PD-inherent specific processing impairment has to be regarded and interpreted under these preliminary aspects.

Methodological differences between the presented studies concern the investigated emotions, the applied tests, as well as calculations. For this reason, it is difficult to determine if STN-DBS selectively impairs the recognition of specific emotions or if it rather leads to an overall deficit. Whereas most studies investigated multiple and different single emotions like disgust, fear, or happiness (e.g., Drapier et al., 2008; Péron et al., 2010a; Mermillod et al., 2014), other studies calculated a general overall emotion score that does not provide information about processing mechanisms for specific emotions (for instance, Schneider et al., 2003; Berney et al., 2007; Péron et al., 2010b; McIntosh et al., 2015). Albuquerque et al. (2014) reported mean values for positive and negative emotions but not for single emotions. Finally, some studies used only a small subset of two facial expressions for their investigation, like happiness and sadness/fear due to experimental settings (Irmen et al., 2017; Martínez-Fernández et al., 2018). Also, Dujardin et al. (2004) decided to eliminate happiness and fear expressions in their setting from the start because of methodological considerations.

Other disparities between studies refer to experimental settings and individual patients’ preconditions. For example, some researchers (e.g., Drapier et al., 2008; Péron et al., 2010a; McIntosh et al., 2015) tested early progression PD patients, that is, patients with motor impairments not past Hoehn and Yahr stage II. Other studies provided data of patients with far more advanced disease progressions (Dujardin et al., 2004; Schroeder et al., 2004). All studies except McIntosh et al. (2015) gave information about patients’ disease duration [with a range from 10.5 ± 3.6 years (Péron et al., 2010b) to 17.0 ± 6.3 years (Schneider et al., 2003)].

Several studies compared emotion processing of facial expressions in PD patients preoperatively and postoperatively, with DBS-OFF results gained before surgery and DBS-ON results assessed several months after surgery (Dujardin et al., 2004; Drapier et al., 2008; Le Jeune et al., 2008; Péron et al., 2010a, b; Albuquerque et al., 2014). All of these except Albuquerque et al. (2014) reported diminished facial emotion (and prosody) recognition after DBS surgery. Albuquerque et al. (2014) did not find any differences between both measure times.

This experimental setting offers the advantage of precluding learning effects of the stimulus material due to long time intervals between both measures. But this time span of at least several months comes along with further subjective, social, and cognitive influences on patients, which cannot be taken into account when interpreting performances and might thus bias the comparability of both testing results. Plus, changes of medication doses between both testing points are common. Moreover, the DBS surgery itself as well as alterations during the operation can have impacts on the performance outcome under DBS ON, which are not present for the OFF condition. Especially Aiello et al. (2014) emphasized the effects of microlesions on facial recognition. They reported diminished facial emotion recognition in patients even before surgery. According to their results, soon after DBS surgery, before turning the stimulator ON, patients were impaired in facial discrimination and recognition tasks for the emotion sadness only but showed recovered ability to recognize disgust. After 4 months post-surgery, patients’ performance in facial emotion recognition remained stable but impaired disgust recognition was prominent again, just like before DBS operation. The authors suggested microlesion effects in the processing of single emotions and stressed the existence of different neural mechanisms for different emotional expressions.

To face the problem of long time intervals and possible diminished comparability of OFF and ON results due to subjective and environmental influences, other studies tested performance only after patients had been operated by switching the stimulator ON and OFF post-operatively. One can assume that possible influences deriving from surgery (microlesions, releasing effects, etc.) have subsided until then and are thus comparable for both testing sessions (DBS-ON and DBS-OFF). However, surgery itself can have a considerable impact on neuropsychological and executive functioning and might provoke alterations in the processing of emotional stimulus material (Brück et al., 2011). Previously, Okun et al. (2009) proposed not STN-DBS itself but rather insertion or lesion effects associated with electrode implantation as a possible underlying mechanism to explain performance differences preoperatively and post-operatively. Reliable comparisons between DBS-treated patients and HC can thus only be made with constraints. Another point that has to be kept in mind when analyzing results of emotion processing under stimulation is the fact that the long-lasting cerebral circuits reorganization following chronic STN-DBS is a long-term procedure and cannot be tackled early after turning the stimulator OFF. Hence, given results in ON/OFF testings rather shortly after DBS surgery do not represent final modulations caused by stimulation; hence, long-term analyses would be desirable in this context. As already mentioned above, general psychiatric anxiety was not associated with impaired emotion perception (Dujardin et al., 2004). However, the sole act of switching the stimulator ON or OFF, respectively, might lead to transient side effects but also subjective inconveniences like bad expectations and worry of experiencing returning (motor) symptoms like tremor in the hands when the stimulation is stopped. This subjectively perceived and testing situation-dependent trouble might also impact patients’ performance and might actually contribute to the differing results concerning facial emotion recognition in the presented studies.

Contrary to the remaining studies reported in this review, Enrici et al. (2017) investigated facial emotion recognition while stimulation was ON but did not test (or did not report) results for DBS-OFF and rather demonstrated comparisons to a group of L-Dopa-treated patients without surgery and to a group of HC. It is hence difficult to draw conclusions about the efficacy and impact of STN-DBS on emotional processing in this study.

Two publications in this review adopt a special position within research of facial emotion recognition under DBS. Irmen et al. (2017) and Martínez-Fernández et al. (2018) did not investigate explicit emotion recognition *per se*, but rather recognition of emotional faces during a Stroop task. Both applied a modified version of the classic Stroop test, the so-called facial emotional Stroop test developed by Etkin et al. (2006). Patients were asked to recognize the emotion that was expressed by faces, while they should ignore the emotion of a word written over these faces. Hence, congruent trials would imply the same emotional valence for both face and word. The Stroop effect refers to the difference in reaction times between congruent and incongruent trials and represents a measure of the overall conflict processing. Martínez-Fernández et al. (2018) examined “happiness,” “fear,” and a neutral condition, whereas Irmen et al. (2017) used “joy” and “grief” in their Stroop test. Irmen et al. (2017) reported no reaction slowing in patients under DBS, thus, demonstrating a defect in within-trial conflict adaptation induced by STN-DBS. Martínez-Fernández et al. (2018) found that STN-DBS, in contrast to levodopa, has no significant impact on emotional conflict processing, but that PD patients suffer from a dysfunction in the early processing of facial emotions, which could be anatomically localized to the inferotemporal cortex and the fusiform gyrus. They further pointed out different regional brain activation, depending on treatment. While levodopa increased activity in areas associated with emotion processing, DBS hypoactivated them. The Stroop effect would hence be modulated by levodopa, but not by STN-DBS, “with this modulation being mainly mediated through the effect of each treatment on the recognition of facial emotion” (Martínez-Fernández et al., 2018).

## Implications

This review summarizes studies investigating the influence of STN-DBS on recognition of emotional facial expressions in PD patients. This summary provides double-sided results. The majority of studies either demonstrated worsening in the processing of at least single specific emotions or reported no changes in facial emotion recognition under STN-DBS. No study was able to show any ameliorations of facial emotion processing in patients when DBS was ON. Such improvements were visible only for non-facial material like written or spoken semantic stimuli and emotional prosody or non-facial pictorial stimuli (Castner et al., 2007; Brück et al., 2011; Serranová et al., 2011; Wagenbreth et al., 2019).

However, these insights are of course biased by the fact that facial emotion processing is already deteriorated in PD patients, even before DBS surgery. Hence, it is rather complicated to define a baseline of facial emotion recognition that refers to “normal” performance in PD patients, and studies investigating DBS impact on this performance always underlie this bias. Furthermore, several methodological as well as clinical and individual factors contribute to different findings and exacerbate a final assertion concerning the efficacy of STN-DBS on facial emotion perception. These factors relate to experimental settings, selection criteria and choice of patients, additional dopaminergic medication next to DBS and its simultaneous intake or suspension, or settings regarding switching the stimulator ON or OFF. A general problem of all studies seems to comprise the recruitment and engagement of patients because all reported STN-DBS studies in this review present rather small sample sizes. With the exception of Albuquerque et al. (2014), who reported results of *N* = 30 patients, no other study was able to present data of more than 24 patients. Indeed, most studies showed results of less than 15 patients. Hence, a difficulty and challenge in the research of STN-DBS effects consist of the availability of data of an appropriate sample size to ensure validity and generalizability.

Finally, further possibly confounding or interacting factors have not been a subject of interest over studies, as for instance gender of patients, different cultural circles, living conditions, and other social factors of patients. For example, it is conceivable that single patients who live rather solitarily and secluded may undergo problems in facial emotion recognition due to inexperience and lack of contact with other people. This might concern particularly “socially and culturally defined” emotions, which were subject of studies assessing not (only) basic emotions but rather emotional gradations or social emotions like the Reading the Mind in the Eyes test (Baron-Cohen et al., 1997). This might be taken into consideration in future investigations.

At any rate, studies demonstrated the possibility of deteriorated recognition of negative emotions out of faces, especially disgust or fear, after DBS surgery. This might have important implications for communication and social living for relatives, nurses, or caregivers of patients. Precautionary arrangements and accords should be entered to ensure optimal cooperation and well-being for patients.

Future studies might further aim to give more attention to the combined investigation of implicit and explicit emotional processing and the DBS influence on both. As this review could demonstrate, there is scarcely STN-DBS research investigating implicit emotional processing because most studies concentrated on emotion processing that is associated with thinking, categorizing, and reflecting.

Finally, it was recently postulated that “what is consistently reported as a group effect seems to be mainly driven by a small, but substantial subgroup of DBS-treated patients” (Højlund et al., 2017; Foki et al., 2018). Effects of treatment may be small and specific to certain individuals. Thus, results should be regarded with respect to interindividual characteristics and intensities as well as to possible heterogeneous gains and losses from DBS.

## Author Contributions

CW, MK, and TZ wrote the main manuscript. H-JH and TZ contributed to conception of the review. All authors reviewed the manuscript, read and approved the final manuscript, and agreed to be accountable for all aspects of the work.

## Conflict of Interest

The authors declare that the research was conducted in the absence of any commercial or financial relationships that could be construed as a potential conflict of interest.

## References

[B1] Abbes M. Lhommée E. Thobois S. Klinger H. Schmitt E. Bichon A. (2018). Subthalamic stimulation and neuropsychiatric symptoms in Parkinson’s disease: results from a long-term follow-up cohort study. *J. Neurol. Neurosurg. Psychiatry* 89 836–843. 10.1136/jnnp-2017-316373 29436490

[B2] Adolphs R. (2002). Recognizing emotions from facial expressions: psychological and neurological mechanisms. *Behav. Cogn. Neurosci. Rev.* 1 21–62. 10.1177/1534582302001001003 17715585

[B3] Adolphs R. Schul R. Tranel D. (1998). Intact recognition of facial emotion in Parkinson’s disease. *Neuropsychology* 12 253–258. 10.1037/0894-4105.12.2.253 9556771

[B4] Aiello M. Eleopra R. Lettieri C. Mondani M. D’Auria S. Belgrado E. (2014). Emotion recognition in Parkinson’s disease after subthalamic deep brain stimulation: differential effects of microlesion and STN stimulation. *Cortex* 51 35–45. 10.1016/j.cortex.2013.11.003 24342106

[B5] Albuquerque L. Coelho M. Martins M. Martins I. P. (2014). STN-DBS does not change emotion recognition in advanced Parkinson’s disease. *Parkinson Relat. Disord.* 20 564–565. 10.1016/j.parkreldis.2014.01.020 24582074

[B6] Alexander G. E. Crutcher M. D. (1990). Functional architecture of basal ganglia circuits: neural substrates of parallel processing. *Trends Neurosci.* 13 266–271. 10.1016/0166-2236(90)90107-l 1695401

[B7] Alexander G. E. DeLong M. R. Strick P. L. (1986). Parallel organization of functionally segregated circuits linking basal ganglia and cortex. *Ann. Rev. Neurosci.* 9 357–381. 10.1146/annurev.neuro.9.1.357 3085570

[B8] Argaud S. Vérin M. Sauleau P. Grandjean D. (2018). Facial emotion recognition in Parkinson’s disease: a review and new hypotheses. *Mov. Disord.* 33 554–567. 10.1002/mds.27305 29473661 PMC5900878

[B9] Assogna F. Pontieri F. Caltagirone C. Spalletta G. (2008). The recognition of facial emotion expressions in Parkinson’s disease. *Eur. Neuropsychopharmacol.* 18 835–848. 10.1016/j.euroneuro.2008.07.004 18707851

[B10] Ballanger B. van Eimeren T. Moro E. Lozano A. M. Hamani C. Boulinguez P. (2009). Stimulation of the subthalamic nucleus and impulsivity: release your horses. *Ann. Neurol.* 66 817–824. 10.1002/ana.21795 20035509 PMC2972250

[B11] Baron-Cohen S. Jolliffe T. Mortimore C. Robertson M. (1997). Another advanced test of theory of mind: evidence from very high functioning adults with autism or asperger syndrome. *J. Child Psychol. Psychiatry* 38 813–822. 10.1111/1469-7610.00715 9363580

[B12] Beatty W. W. Staton R. D. Weir W. S. Monson N. Whitaker H. A. (1989). Cognitive disturbances in Parkinson’s disease. *J. Geriatr. Psychiatry Neurol.* 2 22–33. 2742731 10.1177/089198878900200106

[B13] Berney A. Panisset M. Sadikot A. F. Ptito A. Dagher A. Fraraccio M. (2007). Mood stability during acute stimulator challenge in Parkinson’s disease patients under long-term treatment with subthalamic deep brainstimulation. *Mov. Dis.* 22 1093–1096. 10.1002/mds.21245 17394245

[B14] Berney A. Vingerhoets F. Perrin A. Guex P. Villemure J. G. Burkhard P. R. (2002). Effect on mood of subthalamic DBS for Parkinson’s disease: a consecutive series of 24 patients. *Neurology* 59 1427–1429. 10.1212/01.wnl.0000032756.14298.18 12427897

[B15] Biseul I. Sauleau P. Haegelen C. Trebon P. Drapier D. Raoul S. (2005). Fear recognition is impaired by subthalamic nucleus stimulation in Parkinson’s disease. *Neuropsychologia* 43 1054–1059. 10.1016/j.neuropsychologia.2004.10.006 15769491

[B16] Blonder L. X. Gur R. E. Gur R. C. (1989). The effects of right and left hemiparkinsonism on prosody. *Brain Lang.* 36 193–207. 10.1016/0093-934x(89)90061-8 2920285

[B17] Borg C. Bedoin N. Bogey S. Michael G. A. Poujois A. Laurent B. (2012). Implicit and explicit emotional processing in Parkinson’s disease. *J. Clin. Exp. Neuropsychol.* 34 289–296. 10.1080/13803395.2011.639296 22229340

[B18] Braak H. Braak E. Yilmazer D. de Vos R. A. I. Jansen E. N. H. Bohl J. (1994). Amygdala pathology in Parkinson’s disease. *Acta Neuropathol.* 88 493–500. 7879596 10.1007/BF00296485

[B19] Braak H. Ghebremedhin E. Rüb U. Bratzke H. Del Tredici K. (2004). Stages in the development of Parkinson’s disease-related pathology. *Cell Tissue Res.* 318 121–134. 15338272 10.1007/s00441-004-0956-9

[B20] Braak H. Rüb U. Schultz C. del Tredici K. (2006). Vulnerability of cortical neurons to Alzheimer’s and Parkinson’s diseases. *J. Alzheimer Dis.* 9 35–44. 10.3233/jad-2006-9s305 16914843

[B21] Breitenstein C. Daum I. Ackermann H. (1998). Emotional processing following cortical and subcortical brain damage: contribution of the fronto-striatal circuitry. *Behav. Neurol.* 11 29–42. 10.1155/1998/579029 11568400

[B22] Brück C. Wildgruber D. Kreifelts B. Krüger R. Wächter T. (2011). Effects of subthalamic nucleus stimulation on emotional prosody comprehension in Parkinson’s disease. *Pub. Library Sci. One* 6:e19140 10.1371/journal.pone 0019140 10.1371/journal.pone PMC3084266 21552518

[B23] Calder A. J. Keane J. Manes F. Antoun N. Young A. W. (2000). Impaired recognition and experience of disgust following brain injury. *Nat. Neurosci.* 3 1077–1078. 10.1038/80586 11036262

[B24] Castner J. E. Chenery H. J. Copland D. A. Coyne T. J. Sinclair F. Silburn P. A. (2007). Semantic and affective priming as a function of stimulation of the subthalamic nucleus in Parkinson’s disease. *Brain* 130 1395–1407. 10.1093/brain/awm059 17430981

[B25] Castrioto A. Lhommée E. Moro E. Krack P. (2014). Mood and behavioural effects of subthalamic stimulation in Parkinson’s disease. *Lancet Neurol.* 13 287–305. 10.1016/S1474-4422(13)70294-1 24556007

[B26] Cheung C. C. Lee T. M. Yip J. T. King K. E. Li L. S. (2006). The differential effects of thalamus and basal ganglia on facial emotion recognition. *Brain Cogn.* 61 262–268. 10.1016/j.bandc.2006.01.008 16540222

[B27] Chikama M. McFarland N. R. Amaral D. G. Haber S. N. (1997). Insular cortical projections to functional regions of the striatum correlate with cortical cytoarchitectonic organization in the primate. *J. Neurosci.* 17 9686–9705. 10.1523/jneurosci.17-24-09686.1997 9391023 PMC6573402

[B28] Clark U. S. Neargarder S. Cronin-Golomb A. (2008). Specific impairments in the recognition of emotional facial expressions in Parkinson’s disease. *Neuropsychologia* 46 2300–2309. 10.1016/j.neuropsychologia.2008.03.014 18485422 PMC2491661

[B29] Cohen H. Gagné M. H. Hess U. Pourcher E. (2010). Emotion and object processing in Parkinson’s disease. *Brain Cogn.* 72 457–463. 20167412 10.1016/j.bandc.2010.01.001

[B30] Cummings J. L. (1993). The neuroanatomy of depression. *J. Clin. Psychiatry* 54 (Suppl.), 14–20. 8270593

[B31] Delaveau P. Salgado-Pineda P. Fossati P. Witias T. Azulay J. P. Blin O. (2010). Dopaminergic modulation of the default mode network in Parkinson’s disease. *Eur. Neuropsychopharmacol.* 20 784–792. 10.1016/j.euroneuro.2010.07.001 20674286

[B32] Delaveau P. Salgado-Pineda P. Witjas T. Micallef-Roll J. Fakra E. Azulay J. P. (2009). Dopaminergic modulation of amgydala activity during emotion recognition in patients with Parkinson disease. *J. Clin. Psychopharmacol.* 29 548–554. 10.1097/JCP.0b013e3181bf1c5f 19910719

[B33] Drapier D. Péron J. Leray E. Sauleau P. Biseul I. Drapier S. (2008). Emotion recognition impairment and apathy after subthalamic nucleus stimulation in Parkinson’s disease have separate neural substrates. *Neuropsychologia* 46 2796–2801. 10.1016/j.neuropsychologia.2008.05.006 18579165

[B34] Dujardin K. Blairy S. Defebvre L. Krystkowiak P. Hess U. Blond S. (2004). Subthalamic nucleus stimulation induces deficits in decoding emotional facial expressions in Parkinson’s disease. *J. Neurol. Neurosurg. Psychiatry* 75 202–208. 10.1136/jnnp.2003.013656 14742588 PMC1738891

[B35] Ekman P. Friesen W. (1976). *Pictures of Facial Affect.* PaloAlto, CA: Consulting Psychologists Press.

[B36] Enrici I. Adenzato M. Ardito R. B. Mitkova A. Cavallo M. Zibetti M. (2015). Emotion processing in Parkinson’s disease: a three-level study on recognition, representation and regulation. *PLoS One* 10:e0131470 10.1371/journal.pone.0131470 PMC4482447 26110271

[B37] Enrici I. Mitkova A. Castelli L. Lanotte M. Lopiano L. Adenzato M. (2017). Deep brain stimulation of the subthalamic nucleus does not negatively affect social cognitive abilities of patients with Parkinson’s disease. *Sci. Rep.* 7:9413 10.1038/s41598-017-09737-6 PMC5573348 28842656

[B38] Erwin R. J. Gur R. C. Gur R. E. Skolnick B. Mawhinney-Hee M. Smailies J. (1992). Facial emotion discrimination: I. task construction and behavioral findings in normal subjects. *Psychol. Res.* 42 231–240. 10.1016/0165-1781(92)90115-j 1496055

[B39] Etkin A. Egner T. Peraza D. M. Kandel E. R. Hirsch J. (2006). Resolving emotional conflict: a role for the rostral anterior cingulate cortex in modulating activity in the amygdala. *Neuron* 51 871–882. 10.1016/j.neuron.2006.07.029 16982430

[B40] Fasano A. Romito L. M. Daniele A. Piano C. Zinno M. Bentivoglio A. R. (2010). Motor and cognitive outcome in patients with Parkinson’s disease 8 years after subthalamic implants. *Brain* 133 2664–2676. 10.1093/brain/awq221 20802207

[B41] Foki T. Hitzl D. Pirker W. Novak K. Pusswald G. Lehrner J. (2018). Individual cognitive change after DBS-surgery in Parkinson’s disease patients using reliable change index methodology. *Neuropsychiatry* 32 149–158. 10.1007/s40211-018-0271-4 29767379 PMC6132487

[B42] Fox E. Russo R. Dutton K. (2002). Attentional bias for threat: evidence for delayed disengagement from emotional faces. *Cogn. Emot.* 16 355–379. 10.1080/02699930143000527 18273395 PMC2241753

[B43] Frank M. J. Seeberger L. C. O’Reilly R. C. (2004). By carrot or by stick: cognitive reinforcement learning in parkinsonism. *Science* 306 1940–1943. 10.1126/science.1102941 15528409

[B44] Froming K. Levy M. Schaffer S. Ekman P. (2006). *The Comprehensive Affect Testing System.* Sharpsburg, PA: Psychology Software, Inc.

[B45] Fudge J. L. Breitbart M. A. Danish M. Pannoni V. (2005). Insular and gustatory inputs to the caudal ventral striatum in primates. *J. Comp. Neurol.* 490 101–118. 10.1002/cne.20660 16052493 PMC2474655

[B46] Geday J. Ostergaard K. Gjedde A. (2006). Stimulation of subthalamic nucleus inhibits emotional activation of fusiform gyrus. *Neuroimage* 33 706–714. 10.1016/j.neuroimage.2006.06.056 16959496

[B47] Goldman A. I. Sripada C. S. (2005). Stimulationist models of face-based emotion recognition. *Cognition* 94 193–213. 10.1016/j.cognition.2004.01.005 15617671

[B48] Gray H. M. Tickle-Degnen L. (2010). A meta-analysis of performance on emotion recognition tasks in Parkinson’s disease. *Neuropsychology* 24 176–191. 10.1037/a0018104 20230112

[B49] Harding A. J. Stimson E. Henderson J. M. Halliday G. M. (2002). Clinical correlates of selective pathology in the amygdala of patients with Parkinson’s disease. *Brain* 125 2431–2445. 10.1093/brain/awf251 12390970

[B50] Haynes W. I. Haber S. N. (2013). The organization of prefrontal-subthalamic inputs in primates provides an anatomical substrate for both functional specificity and integration: implications for Basal Ganglia models and deep brain stimulation. *J. Neurosci.* 33 4804–4814. 10.1523/JNEUROSCI.4674-12.2013 23486951 PMC3755746

[B51] Hershey T. Campbell M. C. Videen T. O. Lugar H. M. Weaver P. M. Hartlein J. (2010). Mapping Go – No-Go performance within the subthalamic nucleus region. *Brain* 133 3625–3634. 10.1093/brain/awq256 20855421 PMC2995882

[B52] Hershey T. Revilla F. J. Wernle A. Gibson P. S. Dowling J. L. Perlmutter J. S. (2004). Stimulation of STN impairs aspects of cognitive control in PD. *Neurology* 62 1110–1114. 10.1212/01.wnl.0000118202.19098.10 15079009

[B53] Hess U. Blairy S. (1995). *Set of Emotional Facial Stimuli.* Montreal: University of Quebec at Montreal.

[B54] Hilker R. Voges J. Weisenbach S. Kalbe E. Burghaus L. Ghaemi M. (2004). Subthalamic nucleus stimulation restores glucose metabolism in associative and limbic cortices and in cerebellum: evidence from a FDG-PET study in advanced Parkinson’s disease. *J. Cereb. Blood Flow Metab.* 24 7–16. 10.1097/01.WCB.0000092831.44769.09 14688612

[B55] Hofmann M. J. Kuchinke L. Tamm S. Võ M. L.-H. Jacobs A. M. (2009). Affective processing within 1/10th of a second: high arousal is necessary for early facilitative processing of negative but not positive words. *Cogn. Affect. Behav. Neurosci.* 9 389–397. 10.3758/9.4.389 19897792

[B56] Højlund A. Petersen M. V. Sridharan K. S. Østergaard K. (2017). Worsening of verbal fluency after deep brain stimulation in Parkinson’s disease: a focused review. *Comp. Struct. Biotechnol. J.* 15 68–74. 10.1016/j.csbj.2016.11.003 27994799 PMC5155048

[B57] Ibarretxe-Bilbao N. Junqué C. Tolosa E. Marti M. J. Valldeoriola F. Bargallo N. (2009). Neuroanatomical correlates of impaired decision-making and facial emotion recognition in early Parkinson’s disease. *Eur. J. Neurosci.* 30 1162–1171. 10.1111/j.1460-9568.2009.06892.x 19735293

[B58] Irmen F. Huebl J. Schroll H. Brücke C. Schneider G. H. Hamker F. H. (2017). Subthalamic nucleus stimulation impairs emotional conflict adaptation in Parkinson’s disease. *Soc. Cogn. Affect. Neurosci.* 12 1594–1604. 10.1093/scan/nsx090 28985419 PMC5647801

[B59] Ishai A. Pessoa L. Bikle P. C. Ungerleider L. G. (2004). Repetition suppression of faces is modulated by emotion. *Proc. Natl. Acad. Sci. U.S.A.* 101 9827–9832. 10.1073/pnas.0403559101 15210952 PMC470759

[B60] Jacobs D. H. Shuren J. Bowers D. Heilman K. M. (1995). Emotional facial imagery, perception and expression in Parkinson’s disease. *Neurology* 45 1696–1702. 10.1212/wnl.45.9.1696 7675229

[B61] Kan Y. Kawamura M. Hasegawa Y. Mochizuki S. Nakamura K. (2002). Recognition of emotion from facial, prosodic and written verbal stimuli in Parkinson’s disease. *Cortex* 38 623–630. 10.1016/s0010-9452(08)70026-1 12465672

[B62] Kan Y. Mimura M. Kamijima K. Kawamura M. (2004). Recognition of emotion from moving facial and prosodic stimuli in depressed patients. *J. Neurol. Neurosurg. Psychiatry* 75 1667–1671. 10.1136/jnnp.2004.036079 15548479 PMC1738863

[B63] Kipps C. M. Duggins A. J. McCusker E. A. Calder A. J. (2007). Disgust and happiness recognition correlate with anteroventral insula and amygdala volume respectively in preclinical Huntington’s disease. *J. Cogn. Neurosci.* 19 1206–1217. 10.1162/jocn.2007.19.7.1206 17583995

[B64] Kissler J. Koessler S. (2011). Emotionally positive stimuli facilitate lexical decisions-an ERP study. *Bio. Psychol.* 86 254–264. 10.1016/j.biopsycho.2010.12.006 21184799

[B65] Krack P. Batiz A. Van Blercom N. Chabardes S. Fraix V. Ardouin C. (2003). Five-year follow-up of bilateral stimulation of the subthalamic nucleus in advanced Parkinson’s disease. *N. Engl. J. Med.* 349 1925–1934. 10.1056/nejmoa035275 14614167

[B66] Krebs J. F. Biswas A. Pascalis O. Kamp-Becker I. Remschmidt H. Schwarzer G. (2011). Face processing in children with autism spectrum disorder: independent or interactive processing of facial identity and facial expression? *J. Autism. Dev. Disord.* 41 796–804. 10.1007/s10803-010-1098-4 20839043

[B67] Kuchinke L. Jacobs A. M. Grubich C. Võ M. L.-H. Conrad M. Herrmann M. (2005). Incidental effects of emotional valence in single word processing: an fMRI study. *NeuroImage* 28 1022–1032. 10.1016/j.neuroimage.2005.06.050 16084739

[B68] Kühn A. A. Hariz M. I. Silberstein P. Tisch S. Kupsch A. Schneider G. H. (2005). Activation of the subthalamic region during emotional processing in Parkinson disease. *Neurology* 65 707–713. 10.1212/01.wnl.0000174438.78399.bc 16157903

[B69] Lawrence A. D. Goerendt I. K. Brooks D. J. (2007). Impaired recognition of facial expressions of anger in Parkinson’s disease patients acutely withdrawn from dopamine replacement therapy. *Neuropsychologia* 45 65–74. 10.1016/j.neuropsychologia.2006.04.016 16780901

[B70] Le Jeune F. Péron J. Biseul I. Fournier S. Sauleau P. Drapier S. (2008). Subthalamic nucleus stimulation affects orbitofrontal conrtex in facial emotion recognition: a PET study. *Brain* 131 1599–1608. 10.1093/brain/awn084 18490359 PMC2408938

[B71] Limousin P. Krack P. Pollak P. Benazzouz A. Ardouin C. Hoffmann D. (1998). Electrical stimulation of the subthalamic nucleus in advanced Parkinson’s disease. *New Engl. J. Med.* 339 1105–1111. 9770557 10.1056/NEJM199810153391603

[B72] LoBue V. (2009). More than just a face in the crowd: detection of emotional facial expressions in young children and adults. *Dev. Sci.* 12 305–313. 10.1111/j.1467-7687.2008.00767.x 19143803

[B73] Lundqvist D. Flykt A. Öhman A. (1998). *The Karolinska Directed Emotional Faces – KDEF.* Stockholm: Karolinska Institutet.

[B74] Madeley P. Ellis A. W. Mindham R. H. (1995). Facial expressions and Parkinson’s disease. *Behav. Neurol.* 8 115–119. 10.3233/BEN-1995-8207 24487429

[B75] Mallet L. Schüpbach M. N’Diaye K. Remy P. Bardinet E. Czernecki V. (2007). Stimulation of subterritories of the subthalamic nucleus reveals its role in the integration of the emotional and motor aspects of behavior. *Proc. Natl. Acad. Sci. U.S.A.* 104 10661–10666. 10.1073/pnas.0610849104 17556546 PMC1965569

[B76] Martínez-Fernández R. Kibleur A. Chabardès S. Fraix V. Castrioto A. Lhommée E. (2018). Different effects of levodopa and subthalamic stimulation on emotional conflict in Parkinson’s disease. *Hum. Brain Mapp.* 39 5014–5027. 10.1002/hbm.24341 30259598 PMC6866290

[B77] McDonald S. Flanagan S. Rollins J. Kinch J. (2003). TASIT: a new clinical tool for assessing social perception after traumatic brain injury. *J. Head Trauma Rehabil.* 18 219–238. 10.1097/00001199-200305000-00001 12802165

[B78] McIntosh L. G. Mannavy S. Camalier C. R. Folley B. S. Albritton A. Konrad P. E. (2015). Emotion recognition in early Parkinson’s disease patients undergoing deep brain stimulation or dopaminergic therapy: a comparison to healthy participants. *Front. Aging Neurosci.* 6:349 10.3389/fnagi.2014.00349 PMC4301000 25653616

[B79] Mermillod M. Mondillon L. Rieu I. Devaux D. Chambres P. Auxiette C. (2014). Dopamine replacement therapy and deep brain stimulation of the subthalamic nuclei induce modulation of emotional processes at different spatial frequencies in Parkinson’s disease. *J. Parkinsons Dis.* 4 97–110. 10.3233/JPD-130256 24496100

[B80] Mondillon L. Mermillod M. Musca S. C. Rieu I. Vidal T. Chambres P. (2012). The combined effect of subthalamic nuclei deep brain stimulation and L-dopa increases emotion recognition in Parkinson’s disease. *Neuropsychologia* 50 2868–2879. 10.1016/j.neuropsychologia.2012.08.016 22944002

[B81] Morris J. S. Frith C. D. Perrett D. I. Rowland D. Young A. W. Calder A. J. (1996). A differential neural response in the human amygdala to fearful and happy facial expressions. *Nature* 383 812–815. 10.1038/383812a0 8893004

[B82] Morris J. S. Scott S. K. Dolan R. J. (1999). Saying it with feeling: neural responses to emotional vocalizations. *Neuropsychologia* 37 1155–1163. 10.1016/s0028-3932(99)00015-9 10509837

[B83] Murphy S. T. Zajonc R. B. (1993). Affect, cognition and awareness: affective priming with optimal and suboptimal stimulus exposures. *J. Pers. Soc. Psychol.* 64 723–739. 10.1037/0022-3514.64.5.723 8505704

[B84] Niedenthal P. M. (2007). Embodying emotion. *Science* 316 1002–1005. 10.1126/science.1136930 17510358

[B85] Öhman A. Mineka S. (2001). Fears, phobias and preparedness: toward an evolved module of fear and fear learning. *Psychol. Rev.* 108 483–522. 10.1037/0033-295x.108.3.483 11488376

[B86] Okun M. S. Fernandez H. H. Wu S. S. Kirsch-Darrow L. Bowers D. Bova F. (2009). Cognition and mood in Parkinson’s disease in subthalamic nucleus versus globus pallidus interna deep brain stimulation: the COMPARE trial. *Ann. Neurol.* 65 586–595. 10.1002/ana.21596 19288469 PMC2692580

[B87] Parent A. Hazrati L. N. (1995). Functional anatomy of the basal ganglia. I. the cortico-basal ganglia-thalamo-cortical loop. *Brain Res. Rev.* 20 91–127. 10.1016/0165-0173(94)00007-c 7711769

[B88] Pell M. D. Leonard C. L. (2005). Facial expression decoding in early Parkinson’s disease. *Cogn. Brain Res.* 23 327–340. 10.1016/j.cogbrainres.2004.11.004 15820640

[B89] Péron J. Biseul I. Leray E. Vicente S. Le Jeune F. Drapier S. (2010a). Subthalamic nucleus stimulation affects fear and sadness recognition in Parkinson’s disease. *Neuropsychology* 24 1–8. 10.1037/a0017433 20063943

[B90] Péron J. Le Jeune F. Haegelen C. Dondaine T. Drapier D. Sauleau P. (2010b). Subthalamic nucleus stimulation affects theory of mind network: a PET study in Parkinson’s disease. *PLoS One* 5:e9919. 10.1371/journal.pone.0009919 20360963 PMC2847915

[B91] Péron J. Cekic S. Haegelen C. Sauleau P. Patel S. Drapier D. (2015). Sensory contribution to vocal emotion deficit in Parkinson’s disease after subthalamic stimulation. *Cortex* 63 172–183. 10.1016/j.cortex.2014.08.023 25282055 PMC5101234

[B92] Péron J. Vicente S. Leray E. Drapier S. Drapier D. Cohen R. (2009). Are dopaminergic pathways involved in theory of mind? a study in Parkinson’s disease. *Neuropsychologia* 47 406–414. 10.1016/j.neuropsychologia.2008.09.008 18845171

[B93] Phillips M. L. Young A. W. Scott S. K. Calder A. J. Andrew C. Giampietro V. (1998). Neural responses to facial and vocal expressions of fear and disgust. *Proc. R. Soc. Bio. Sci.* 265 1809–1817. 10.1098/rspb.1998.0506 9802236 PMC1689379

[B94] Phillips M. L. Young A. W. Senior C. Brammer M. Andrew C. Calder A. J. (1997). A specific neural substrate for perceiving facial expressions of disgust. *Nature* 389 495–498. 10.1038/39051 9333238

[B95] Robert G. Le Jeune F. Lozachmeur C. Drapier S. Dondaine T. Péron J. (2012). Apathy in patients with Parkinson disease without dementia or depression: a PET study. *Neurology* 79 1155–1160. 10.1212/WNL.0b013e3182698c75 22895582

[B96] Roca M. Torralva T. Gleichgerrcht E. Chade A. Arévalo G. G. Gershanik O. (2010). Impairments in social cognition in early medicated and unmedicated Parkinson disease. *Cogn. Behav. Neurol.* 23 152–158. 10.1097/wnn.0b013e3181e078de 20829664

[B97] Romito L. Raja M. Daniele A. Contarino M. F. Bentivoglio A. R. Barbier A. (2002). Transient mania with hypersexuality after surgery for high frequency stimulation of the subthalamic nucleus in Parkinson’s disease. *Mov. Disord.* 17 1371–1374. 10.1002/mds.10265 12465087

[B98] Ruffman T. Henry J. D. Livingstone V. Phillips L. H. (2008). A meta-analytic review of emotion recognition and aging: implications for neuropsychological models of aging. *Neurosci. Biobehav. Rev.* 32 863–881. 10.1016/j.neubiorev.2008.01.001 18276008

[B99] Sander D. Grandjean D. Pourtois G. Schwartz S. Seghier M. L. Scherer K. R. (2005). Emotion and attention interactions in social cognition: brain regions involved in processing anger prosody. *Neuroimage* 28 848–858. 10.1016/j.neuroimage.2005.06.023 16055351

[B100] Sato W. Yoshikawa S. Kochiyama T. Matsumura M. (2004). The amygdala processes the emotional significance of facial expressions: an fMRI investigation using the interaction between expression and face direction. *Neuroimage* 22 1006–1013. 10.1016/j.neuroimage.2004.02.030 15193632

[B101] Schneider F. Habel U. Volkmann J. Regel S. Kornischka J. Sturm V. (2003). Deep brain stimulation of the subthalamic nucleus enhances emotional processing in Parkinson disease. *Arch. Gen. Psychiatry* 60 296–302. 10.1001/archpsyc.60.3.296 12622663

[B102] Schröder C. Möbes J. Schütze M. Szymanowski F. Nager W. Bangert M. (2006). Perception of emotional speech in Parkinson’s disease. *Mov. Disord.* 21 1774–1778. 10.1002/mds.21038 16830324

[B103] Schroeder U. Kuehler A. Haslinger B. Erhard P. Fogel W. Tronnier V. M. (2002). Subthalamic nucleus stimulation affects striato-anterior cingulate cortex circuit in a response conflict task: a PET study. *Brain* 125 1995–2004. 10.1093/brain/awf199 12183345

[B104] Schroeder U. Kuehler A. Hennenlotter A. Haslinger B. Tronnier V. M. Krause M. (2004). Facial expression recognition and subthalamic nucleus stimulation. *J. Neurol. Neurosurg. Psychiatry* 75 648–650. 10.1136/jnnp.2003.019794 15026519 PMC1739017

[B105] Schroeder U. Kuehler A. Lange K. W. Haslinger B. Tronnier V. M. Krause M. (2003). Subthalamic nucleus stimulation affects a frontotemporal network: a PET study. *Ann. Neurol.* 54 445–450. 10.1002/ana.10683 14520655

[B106] Serranová T. Jech R. Dusek P. Sieger T. Ruzicka F. Urgosik D. (2011). Subthalamic nucleus stimulation affects incentive salience attribution in Parkinson’s disease. *Mov. Disord.* 26 2260–2266. 10.1002/mds.23880 21780183

[B107] Sprengelmeyer R. Young A. W. Calder A. J. Karnat A. Lange H. Hömberg V. (1996). Loss of disgust. perception of faces and emotions in huntington’s disease. *Brain* 119 1647–1665. 10.1093/brain/119.5.1647 8931587

[B108] Sprengelmeyer R. Young A. W. Mahn K. Schroeder U. Woitalla D. Büttner T. (2003). Facial expression recognition in people with medicated and unmedicated Parkinson’s disease. *Neuropsychologia* 41 1047–1057. 10.1016/s0028-3932(02)00295-6 12667540

[B109] Susa G. Pitică I. Benga O. Miclea M. (2012). The self regulatory effect of attentional control in modulating the relationship between attentional biases toward threat and anxiety symptoms in children. *Cogn. Emot.* 26 1069–1083. 10.1080/02699931.2011.638910 22404477

[B110] Suzuki A. Hoshini T. Shigemasu K. Kawamura M. (2006). Disgust specific impairment of facial expression recognition in Parkinson’s disease. *Brain* 129 707–717. 10.1093/brain/awl011 16415306

[B111] Temel Y. Blokland A. Steinbusch H. W. Visser-Vandewalle V. (2005). The functional role of the subthalamic nucleus in cognitive and limbic circuits. *Prog. Neurobiol.* 76 393–413. 10.1016/j.pneurobio.2005.09.005 16249050

[B112] Tessitore A. Hariri A. R. Fera F. Smith W. G. Chase T. N. Hyde T. M. (2002). Dopamine modulates the response of the human amgydala: a study in Parkinson’s disease. *J. Neurosci.* 22 9099–9103. 10.1523/jneurosci.22-20-09099.2002 12388617 PMC6757686

[B113] Tottenham N. Tanaka J. W. Leon A. C. McCarry T. Nurse M. Hare T. A. (2009). The NimStim set of facial expressions: judgments from untrained research participants. *Psychiatry Res.* 168 242–249. 10.1016/j.psychres.2008.05.006 19564050 PMC3474329

[B114] Trautmann S. A. Fehr T. Herrmann M. (2009). Emotions in motion: dynamic compared to static facial expressions of disgust and happiness reveal more widespread emotion-specific activations. *Brain Res.* 1284 100–115. 10.1016/j.brainres.2009.05.075 19501062

[B115] Vicente S. Biseul I. Péron J. Philippot P. Drapier S. Drapier D. (2009). Subthalamic nucleus stimulation affects subjective emotional experience in Parkinson’s disease patients. *Neuropsychologia* 47 1928–1937. 10.1016/j.neuropsychologia 19428425

[B116] Vizcarra J. A. Situ-Kcomt M. Artusi C. A. Duker A. P. Lopiano L. Okun M. S. (2019). Subthalamic deep brain stimulation and levodopa in Parkinson’s disease: a meta-analysis of combined effects. *J. Neurol.* 266 289–297. 10.1007/s00415-018-8936-2 29909467

[B117] Wagenbreth C. Kuehne M. Voges J. Heinze H. J. Zaehle T. (2019). Deep brain stimulation of the subthalamic nucleus selectively modulates emotion recognition of facial stimuli in Parkinson’s patients. *J. Clin. Med.* 8:E1335 10.3390/jcm8091335 PMC6781243 31466414

[B118] Wagenbreth C. Rieger J. Heinze H. J. Zaehle T. (2014). Seeing emotions in the eyes – inverse priming effects induced by eyes expressing mental states. *Front. Psychol.* 5:1039. 10.3389/fpsyg.2014.01039 25278925 PMC4166113

[B119] Wagenbreth C. Wattenberg L. Heinze H. J. Zaehle T. (2016). Implicit and explicit processing of emotional facial expressions in Parkinson’s disease. *Behav. Brain Res.* 303 182–190. 10.1016/j.bbr.2016.01.059 26850933

[B120] Wagenbreth C. Zaehle T. Galazky I. Voges J. Guitart-Masip M. Heinze H. J. (2015). Deep brain stimulation of the subthalamic nucleus modulates reward processing and action selection in Parkinson patients. *J. Neurol.* 262 1541–1547. 10.1007/s00415-015-7749-9 25929662

[B121] Wentura D. Rothermund K. Bak P. (2000). Automatic vigilance: the attention-grabbing power of approach- and avoidance-related social information. *J. Pers. Soc. Psychol.* 78 1024–1037. 10.1037//0022-3514.78.6.1024 10870906

[B122] Wieser M. J. Muhlberger A. Alpers G. W. Macht M. Ellgring H. Pauli P. (2006). Emotion processing in Parkinson’s disease: dissociation between early neuronal processing and explicit ratings. *Clin. Neurophysiol.* 117 94–102. 10.1016/j.clinph.2005.09.009 16330254

[B123] Wildgruber D. Ackermann H. Kreifelts B. Ethofer T. (2006). Cerebral processing of linguistic and emotional prosody: fMRI studies. *Prog. Brain Res.* 156 249–268. 10.1016/S0079-6123(06)56013-3 17015084

[B124] Xi C. Zhu Y. Mu Y. Chen B. Dong B. Cheng H. (2015). Theory of mind and decision-making processes are impaired in Parkinson’s disease. *Behav. Brain Res.* 279 226–233. 10.1016/j.bbr.2014.11.035 25435317

[B125] Yip J. T. H. Lee T. M. C. Ho S. L. Tsang K. L. Li L. S. W. (2003). Emotion recognition in patients with Parkinson’s Disease. *Mov. Disord.* 18 1115–1122. 10.1002/mds.10497 14534914

[B126] Yoshimura N. Kawamura M. Masaoka Y. Homma I. (2005). The amygdala of patients with Parkinson’s disease is silent in response to fearful facial expressions. *Neuroscience* 131 523–534. 10.1016/j.neuroscience.2004.09.054 15708493

[B127] Young A. W. Perrett D. Calder A. Sprengelmeyer R. H. Ekman P. (2002). *Facial Expressions of Emotions: Stimuli and Test (FEEST).* Thurstone: Thames Valley Test Company.

[B128] Zaehle T. Wagenbreth C. Voges J. Heinze H. J. Galazky I. (2017). Effects of deep brain stimulation of the subthalamic nucleus on perceptual decision making. *Neuroscience* 343 140–146. 10.1016/j.neuroscience.2016.11.044 27956065

